# Multi-modal low-dose medical imaging through instruction-guided unified AI

**DOI:** 10.3389/fmed.2026.1691143

**Published:** 2026-01-16

**Authors:** Hengliang Lang, Yanjun Zhou, Yibo Yu, Zhaoyin Su, Huixue Zhuge, Weitao Wang, Ding Fang, Jiaji Qin, Min Wei, Rubing Lin, Chao Li

**Affiliations:** 1 Department of Emergency, The Brain Hospital of Guangxi Zhuang Autonomous Region, Liuzhou, China; 2 Shenzhen Institute of Advanced Technology, Chinese Academy of Sciences, Shenzhen, China; 3 The First School of Clinical Medicine, Lanzhou University, Lanzhou, China; 4 Department of Orthopedics, Shenzhen Children's Hospital, Shenzhen, China

**Keywords:** computer vision, CT, medical image, MRI, neural network, PET, radiology, restoration

## Abstract

**Background:**

Ionizing radiation from PET/CT warrants dose reduction. However, lowering dose can degrade image quality and affect diagnosis. Many machine-learning approaches exist. Nevertheless, most are built for a single task and are difficult to deploy across multi-modal workflows. We sought to develop and evaluate a unified model that handles common restoration tasks across modalities.

**Methods:**

We developed the Multi-modal Instruction-guided Restoration Architecture (MIRA-Net), a U-Net–based framework with an adaptive guidance module. The module estimates modality and degradation indicators from the input and produces a low-dimensional instruction that modulates feature processing throughout the network, selecting task-appropriate pathways within a single model. Performance was assessed on CT denoising, PET synthesis, and MRI super-resolution. Additionally, a double-blind reader study was conducted with board-certified radiologists.

**Results:**

Trained on individual tasks, MIRA-Net matched or exceeded strong task-specific baselines. When trained as a single unified model across CT, PET, and MRI, it maintained comparable performance without a meaningful drop from single-task training. Local clinical dataset validation demonstrated robust generalization with consistent performance metrics. In the reader study, MIRA-Net outputs were more often judged diagnostic and received higher scores for anatomical clarity, lesion conspicuity, and noise control.

**Conclusion:**

MIRA-Net provides a high-fidelity solution for multi-modal medical image restoration. Its instruction-guided architecture successfully mitigates task interference, demonstrating an effective pathway to reducing radiation exposure without sacrificing diagnostic quality.

## Introduction

Medical imaging stands as a cornerstone of modern clinical diagnostics, demonstrably saving over 40,000 lives annually and delivering profound systemic benefits (1, 2). This technology saves medical experts 1.8 billion work-hours annually and generates over €200 billion in healthcare cost savings (3). Over the past decade, the demand for medical imaging has surged, particularly in the United States. There has been a substantial increase in the utilisation of advanced modalities such as CT, MRI, and PET—the last of which has seen a growth rate exceeding 100% (4). However, despite its immense clinical value, hybrid imaging modalities like PET/CT introduce significant radiation safety concerns, exposing patients to high levels of ionizing radiation far exceeding natural background levels (5, 6). This issue is particularly acute for patient cohorts such as paediatric epilepsy patients (7), who often require multiple PET/CT scans in short succession for precise lesion localisation, accumulating a radiation dose that poses a risk of irreversible damage to developing organs and tissues (8). Consequently, a paramount challenge in nuclear and radiological medicine is to substantially reduce the patient’s radiation burden without compromising diagnostic image fidelity.

Artificial intelligence (AI), particularly image processing algorithms based on deep neural networks, has emerged as a revolutionary technology in the field of low-dose medical imaging (9). In recent years, advanced neural architectures have been successfully employed to reduce PET tracer doses and CT radiation exposure while maintaining image quality comparable to full-dose protocols. These include Generative Adversarial Networks (10, 11), Denoising Diffusion Probabilistic Models (12–14), and Convolutional Neural Networks (15, 16), which are adept at learning hierarchical spatial features through local receptive fields and weight sharing. Among these, the UNet encoder-decoder architecture has become a standard in medical image segmentation and restoration due to its effective multi-scale representation and feature extraction capabilities (17, 18). However, CT imaging faces persistent challenges including radiation dose optimization, metal artifacts, and noise amplification in low-dose protocols (19, 20), while MRI contends with long acquisition times, motion artifacts, and limited spatial resolution in rapid scanning sequences (21–23). These technological advances, however, confront a fundamental limitation: the majority of existing models are single-function systems optimised for a specific medical imaging task. Their performance degrades significantly when applied to scenarios beyond their original design scope (24). In the context of modern multi-modal imaging, where multiple image quality enhancement tasks must be performed concurrently, such single-task models struggle to address the inherent data distribution differences between modalities (25). Deploying a separate model for each task escalates system complexity, diminishes resource utilisation efficiency, and increases maintenance overhead in clinical practice (26).

To address these challenges, we have developed a breakthrough solution that overcomes the inherent limitations of single-task models. The challenges in this domain extend beyond the diversity of input degradation patterns to the intrinsic disparities in data distribution across different imaging modalities. These fundamental inter-modality differences create a performance bottleneck for conventional multi-modal architectures. Here, we introduce the Multi-modal Instruction-guided Restoration Architecture (MIRA-Net), a unified framework specifically designed to resolve this conflict. The core innovation of MIRA-Net is an intelligent guidance mechanism that first identifies the modality of the input image and then adaptively routes it to the most appropriate, specialized processing branch within a single, unified structure. In contrast to prompt-driven restoration, which typically relies on an externally provided task token or human-specified prompt, MIRA-Net operates in a blind setting: ARC infers a compact instruction directly from the corrupted image. Moreover, unlike hard dynamic-routing or mixture-of-experts designs that select discrete expert branches, our guidance is applied continuously via layer-wise feature modulation, enabling task-adaptive behavior without proliferating modality-specific subnetworks.

## Materials and methods

### Data sources

Our unified medical image restoration framework addresses three clinically relevant tasks—MRI super-resolution reconstruction, CT denoising, and PET image synthesis. A comprehensive summary of dataset composition, preprocessing procedures, and experimental splits for each task is provided in Supplementary Table S1.

For MRI super-resolution reconstruction, we utilized 578 high-quality T2-weighted MRI scans from the public IXI database. From each 3D volumetric data, we extracted the central 100 two-dimensional slices, effectively avoiding signal instabilities and incomplete anatomical structures in peripheral regions. Low-quality images were simulated through 4-fold k-space undersampling, mimicking fast acquisition scenarios in clinical practice. The final dataset was divided into training, validation, and testing sets in a 405:59:114 ratio, ensuring reliable model evaluation.

For CT denoising, experiments were conducted using the 2016 NIH AAPM-Mayo Clinic Low-Dose CT Grand Challenge dataset (27). This dataset comprises paired standard-dose high-quality CT images and quarter-dose low-quality CT images, providing noise distribution characteristics representative of real clinical environments. The experimental data was scientifically allocated in an 8:1:1 ratio for training, validation, and testing.

For PET image synthesis, experimental data were acquired using a PolarStar m660 PET/CT system with 293 MBq of ^18^F-FDG tracer, yielding 159 high-quality PET images. To simulate low-dose acquisition, we implemented a 12-fold dose reduction through list-mode random sampling while maintaining consistent acquisition geometry. Both high and low-quality images were reconstructed using the standard OSEM method (28), ensuring processing consistency. Each PET dataset, was segmented into 192 two-dimensional slices. After removing pure air layers containing no effective information, the final dataset was divided into training, validation, and testing sets in a 120:10:29 ratio.

To further evaluate the model’s generalizability and clinical applicability, additional validation was conducted using locally acquired clinical datasets, including 142 paired CT scans and 89 brain PET studies with institutional approval. All patient data were anonymized to protect privacy. Detailed information of these datasets is presented in Supplementary Table S1.

### Network architecture

**Figure 1:**
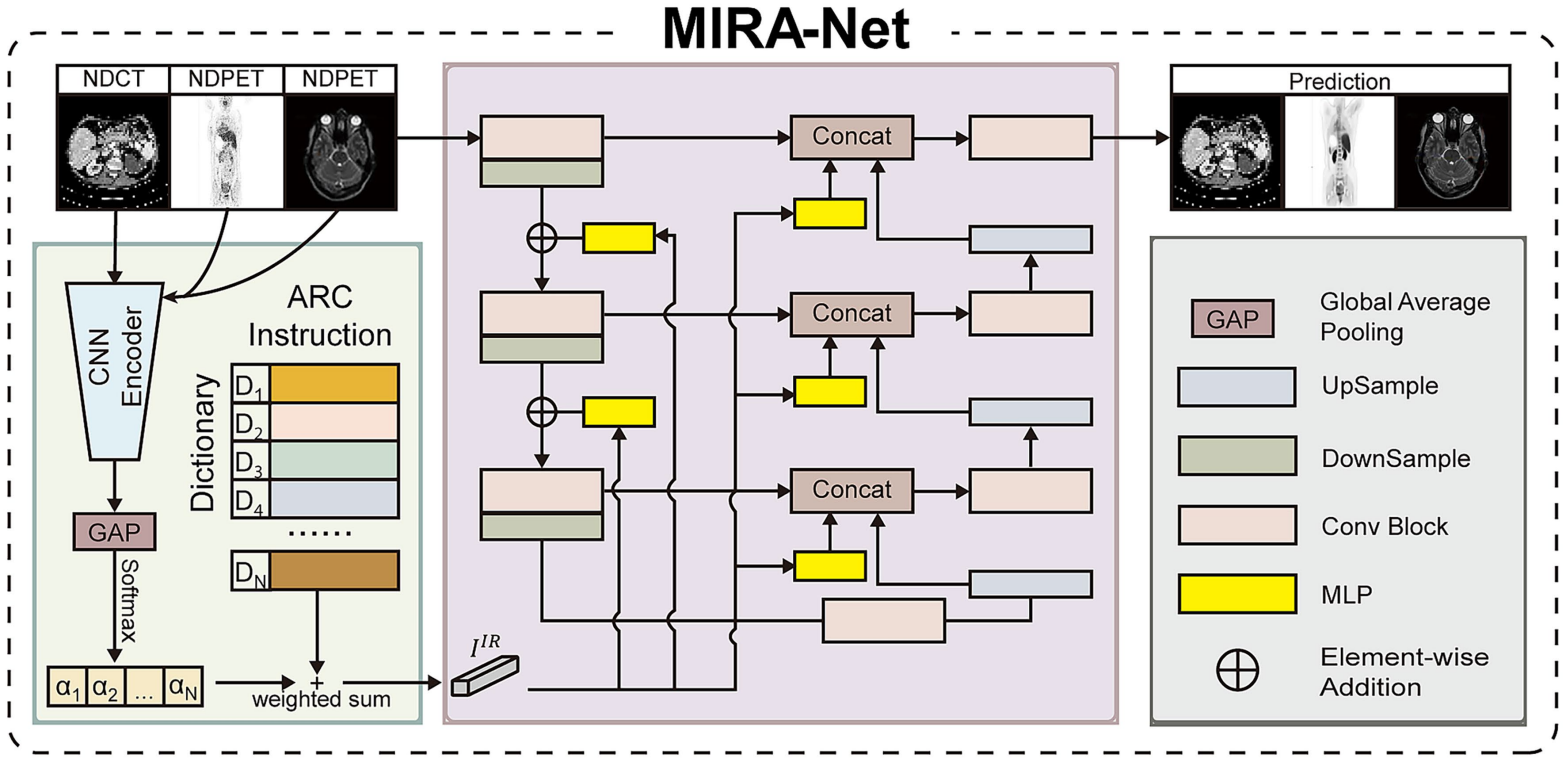
Architecture of the proposed MIRA-Net framework.

Developing a unified model capable of addressing diverse medical image restoration tasks presents fundamental challenges in computational imaging. The primary difficulty lies in the inherent interference between disparate task objectives when processed within a single network architecture. To address this challenge, we propose MIRA-Net, a unified framework built upon the U-Net architecture, as illustrated in Figure 1. The core innovation of our approach is the Adaptive Restoration Core, a novel module that dynamically generates task-specific guidance vectors directly from input image characteristics.

The proposed architecture processes a low-quality input image $I^{\mathrm{LQ}}\in {\mathbb{R}}^{H\times W\times 1}$ through an initial 3×3 convolutional layer to extract shallow features $I^{\mathrm{SF}}$ . These features are subsequently processed by a hierarchical encoder comprising standard convolutional blocks, yielding deep feature representations $I^{\mathrm{DF}}$ . To address the inherent complexities of multi-task restoration, the ARC module analyzes input characteristics and generates a task-specific instruction vector $I^{\mathrm{IR}}$ . As summarized in Equations 1–3, this adaptive process operates in an unsupervised manner according to:

$$I^{\mathrm{IR}}=\sum_{i=1}^{N}\alpha_{i}D_{i},\;\;\text{where}\;\;\alpha =\text{Softmax}(\mathrm{GAP}(E(I^{\mathrm{LQ}})))$$ (1)

where ℰ(⋅) denotes a compact three-layer CNN encoder that evaluates the degradation characteristics of the input image $I^{\mathrm{LQ}}$ . The encoder output is aggregated through global average pooling (GAP) and subsequently transformed into a probability distribution $\alpha ={\left[\alpha_{1},\alpha_{2},\ldots ,\alpha_{N}\right]}^{T}$ via the Softmax function. These attention weights dynamically select and linearly combine primitive restoration strategies from a learnable dictionary $D=[D_{1},D_{2,}\ldots ,D_{N}]\in {\mathbb{R}}^{256\times N}$ , where each column vector $D_{i}\in {\mathbb{R}}^{256}$ encodes a fundamental restoration operation. The resulting instruction vector $I^{\mathrm{IR}}\in {\mathbb{R}}^{256}$ provides task-specific guidance tailored to the input degradation pattern.

### Adaptive feature modulation mechanism

The effectiveness of MIRA-Net relies on the mechanism through which the instruction vector $I^{\mathrm{IR}}$ guides the network’s computational flow. We implement an adaptive feature modulation strategy that translates the global instruction into localized, layer-specific transformations. Each convolutional block within both the encoder and decoder pathways incorporates a dedicated multi-layer perceptron (MLP) that processes the global instruction vector $I^{\mathrm{IR}}$ and generates affine transformation parameters: a scaling vector γ and a bias vector β .

$$\left[\gamma ,\beta ]=\mathrm{MLP}(I^{\mathrm{IR}}\right)$$ (2)

These parameters modulate the feature maps within each block through channel-wise affine transformations that recalibrate the feature representations:

$$F_{\mathrm{mod}}=\gamma ⊙F_{\text{orig}}+\beta$$ (3)

where $F_{\text{orig}}$ and $F_{\mathrm{mod}}$ denote the original and modulated feature maps, respectively, and ⊙ represents element-wise multiplication.

### Evaluation metrics

To comprehensively evaluate the performance of our proposed MIRA-Net framework, we employed three widely-accepted quantitative metrics in medical image restoration tasks. Peak Signal-to-Noise Ratio (PSNR) measures the ratio between the maximum possible power of a signal and the power of corrupting noise, providing an objective assessment of reconstruction quality where higher values indicate better image fidelity (29). Structural Similarity Index Measure (SSIM) evaluates the structural information preservation between the restored and reference images by considering luminance, contrast, and structural components, with values closer to 1.0 representing superior perceptual quality (30). Root Mean Square Error (RMSE) quantifies the pixel-wise differences between the restored and ground truth images, where lower values demonstrate better restoration accuracy (31).

### Statistical analysis

All quantitative comparisons of PSNR, SSIM, and RMSE were evaluated with paired two-sided t-tests against MIRA-Net outputs on a per-case basis. *p*-values are reported in Supplementary Tables S2, S3 and interpreted with a Bonferroni-adjusted significance threshold of *α* = 0.0167 for the three comparator models per modality. Reader-study Likert scores were analyzed with the Wilcoxon signed-rank test versus the corresponding low-dose inputs, with Bonferroni correction across modalities. Inter-reader agreement across the three radiologists was quantified using Fleiss’ kappa (*κ* = 0.82).

## Result

### Single task

To establish a baseline, we benchmarked MIRA-Net against state-of-the-art task-specific models across three medical image restoration tasks. As shown in Supplementary Table S2, MIRA-Net consistently demonstrates superior or competitive performance across all tasks. Specifically, in 4 × MRI super-resolution, MIRA-Net achieved a PSNR of 31.7824 and an SSIM of 0.9358, exceeding Restormer and SwinIR. In low-dose CT denoising, it reached a PSNR of 33.4872, outperforming all comparators. In 12 × dose-reduction PET image synthesis, MIRA-Net delivered a PSNR of 36.9445 and an SSIM of 0.9432, surpassing recent architectures such as SpachTransformer. Paired t-tests against MIRA-Net confirmed that the differences were statistically significant (MRI: SRCNN *p* = 0.0008, VDSR *p* = 0.012, SwinIR *p* = 0.041; CT: CNN *p* = 0.0065, REDCNN *p* = 0.018, Eformer *p* = 0.027; PET: ContextCNN *p* = 0.0031, DCNN *p* = 0.0095, ARGAN *p* = 0.021), indicating that the PSNR/SSIM improvements are unlikely due to chance.

### Unified multi-task performance

To validate the fundamental advantage of our unified framework, we evaluated MIRA-Net’s performance under a unified multi-task training paradigm. Supplementary Table S3 presents a comprehensive comparison of MIRA-Net against competing unified architectures, including AirNet (32), Eformer (33), Spach Transformer (34) and DRMC (35). Across all three modalities, paired comparisons in Supplementary Table S3 yield *p*-values <0.05 for every competing method versus MIRA-Net, confirming that the multi-task gains are statistically significant. Specifically, compared to the second-best performer, Spach Transformer, MIRA-Net achieves superior performance in MRI super-resolution (31.4567 vs. 31.1799 dB) and CT denoising (33.5024 vs. 33.4740 dB), while remaining competitive in PET synthesis (36.8124 vs. 36.7547 dB). Against DRMC, MIRA-Net shows significant advantages across all modalities: +1.91 dB in MRI (31.4567 vs. 29.5466 dB), +0.23 dB in CT (33.5024 vs. 33.2770 dB), and +0.62 dB in PET (36.8124 vs. 36.1909 dB). Notably, MIRA-Net delivers significant improvements over AirNet, a representative parameter-sharing unified model, with gains of +0.81 dB in MRI, +1.20 dB in CT, and +0.90 dB in PET. Furthermore, MIRA-Net consistently outperforms all competitors in SSIM metrics, demonstrating superior structural preservation.

**Figure 2:**
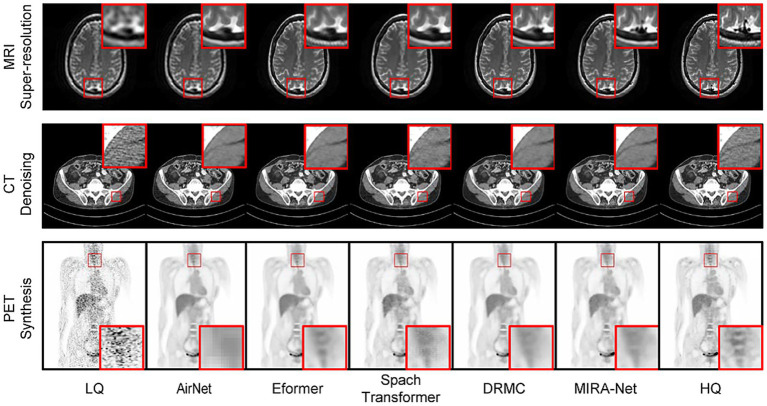
Visual comparison of medical image restoration results across three modalities. Red boxes highlight regions of interest.

These quantitative advantages are visually corroborated in Figure 2. The magnified regions show that for MRI super-resolution, MIRA-Net recovers finer and sharper brain-tissue textures that most closely approximate the high-quality (HQ) reference images. In the CT denoising task, MIRA-Net optimally preserves tissue boundaries and internal structures while effectively removing noise, avoiding the blurring and artifacts produced by other methods. Similarly, for PET synthesis, the images generated by MIRA-Net exhibit the clearest delineation and highest contrast of lesions with effective background noise suppression, demonstrating superior visual fidelity.

### Feature space analysis

**Figure 3:**
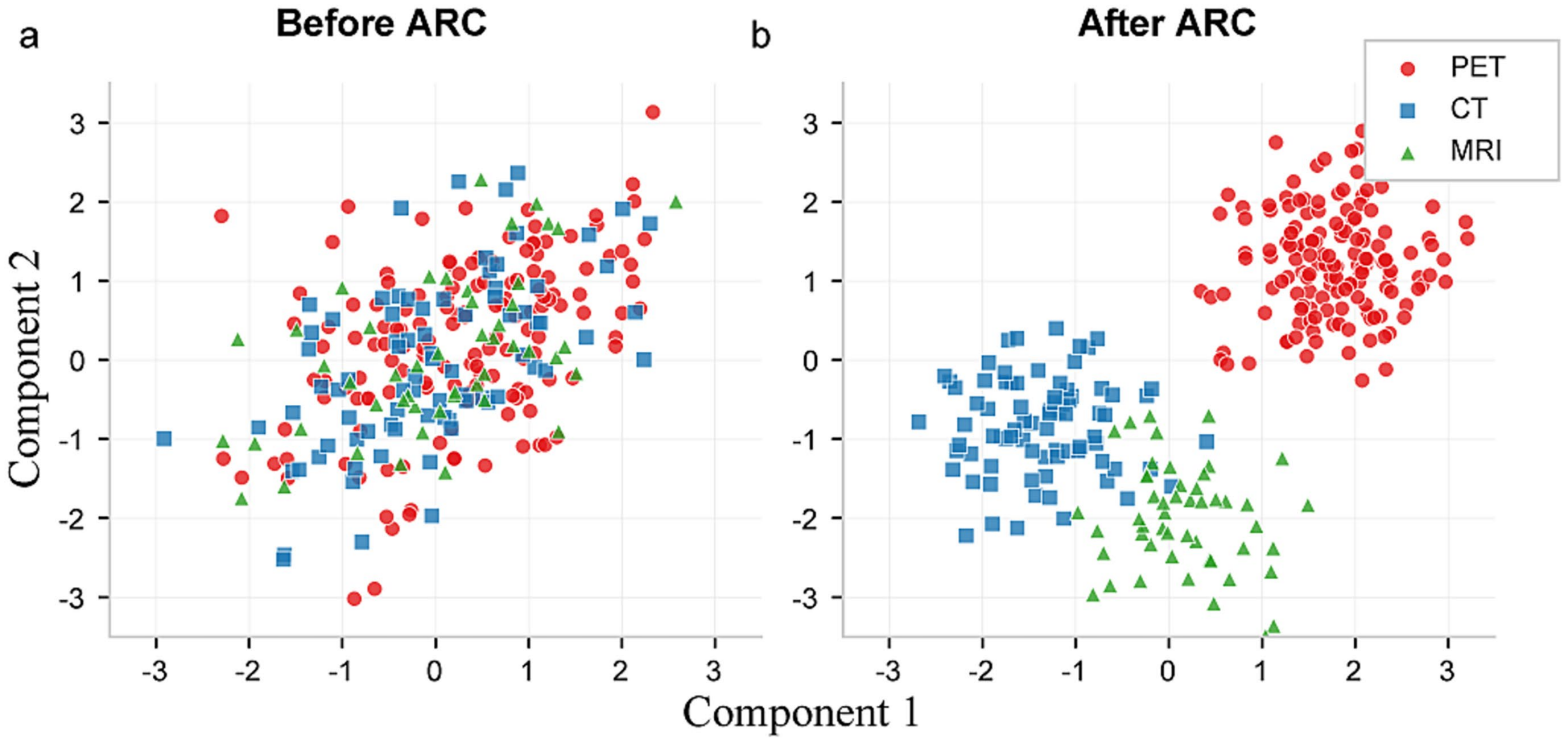
*t*-SNE visualization of feature space distributions for multi-modal medical imaging. **(a)** Feature representations before applying the ARC module. **(b)** Feature representations after applying the ARC module.

The effectiveness of our ARC module in enabling modality-specific processing is demonstrated through t-SNE visualization analysis (Figure 3). Before algorithm training, feature representations from different medical imaging modalities exhibit significant overlap and entanglement in the feature space, making it challenging for conventional unified models to distinguish between different imaging tasks. However, after incorporating the ARC-guided training paradigm, the feature distributions become remarkably well-separated, with each modality forming distinct clusters in the embedding space.

### Generalization to local clinical datasets

**Figure 4:**
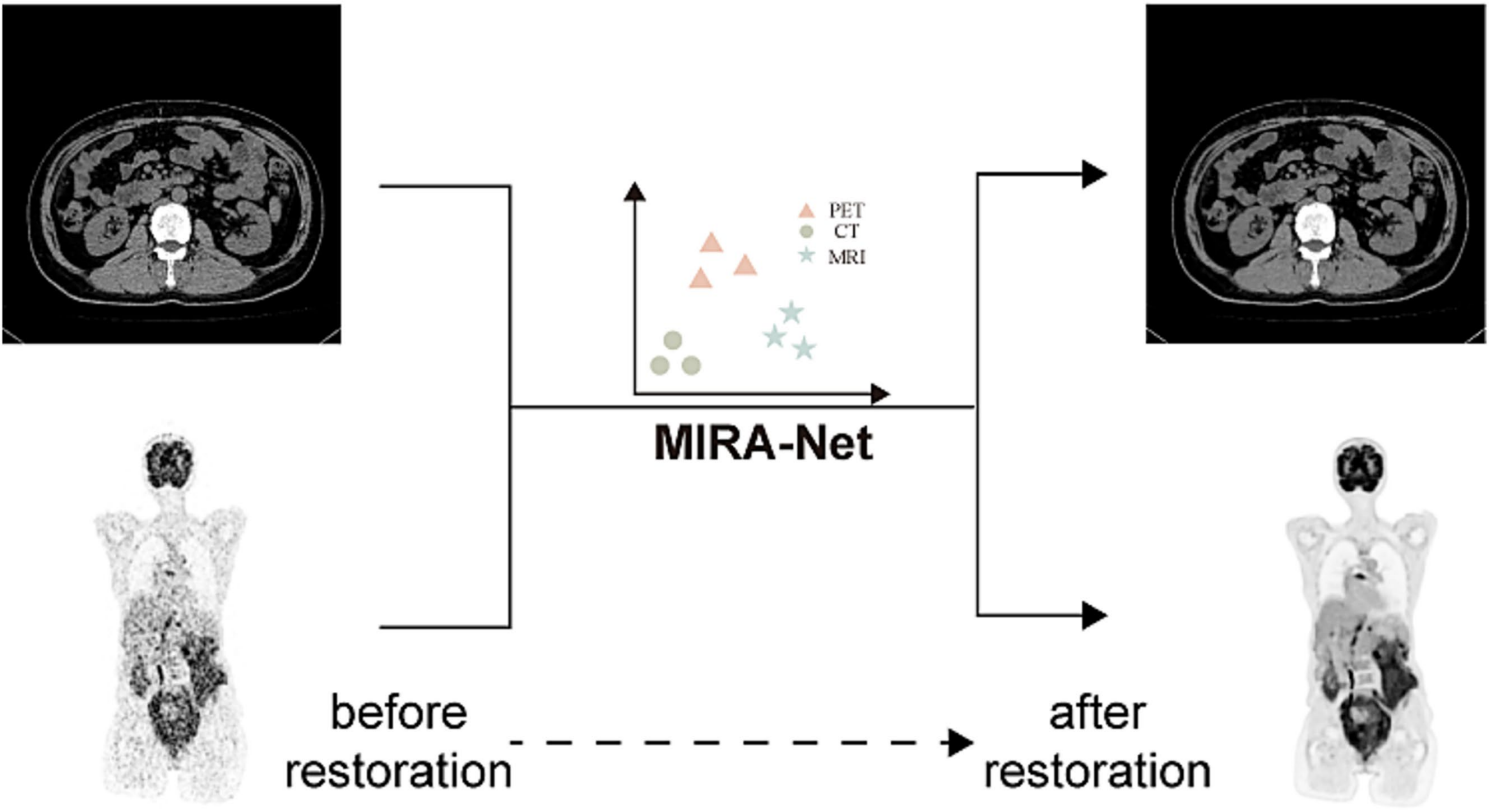
MIRA-Net restoration performance on local clinical CT and PET datasets. Before restoration (left) and after restoration (right) images.

To assess the real-world generalization capability of MIRA-Net, we evaluated the trained model on our locally acquired clinical datasets. As shown in Figure 4, for the local CT dataset, MIRA-Net achieved a PSNR of 58.81 dB and SSIM of 0.0.9913. Similarly, for the local PET dataset, MIRA-Net delivered consistent performance with a PSNR of 38.17 dB and SSIM of 0.9514.

### Clinical validation and diagnostic utility

To ascertain the clinical significance and diagnostic utility of our framework, we conducted a rigorous double-blind study involving two independent, board-certified radiologists with 8, 12, and 20 years of clinical experience, respectively. They assessed the quality of restored CT, PET, and MRI images using a standardized 5-point Likert scale across three criteria: anatomical clarity, lesion conspicuity, and image noise. The detailed evaluation criteria are provided in Supplementary Table S4.

**Figure 5:**
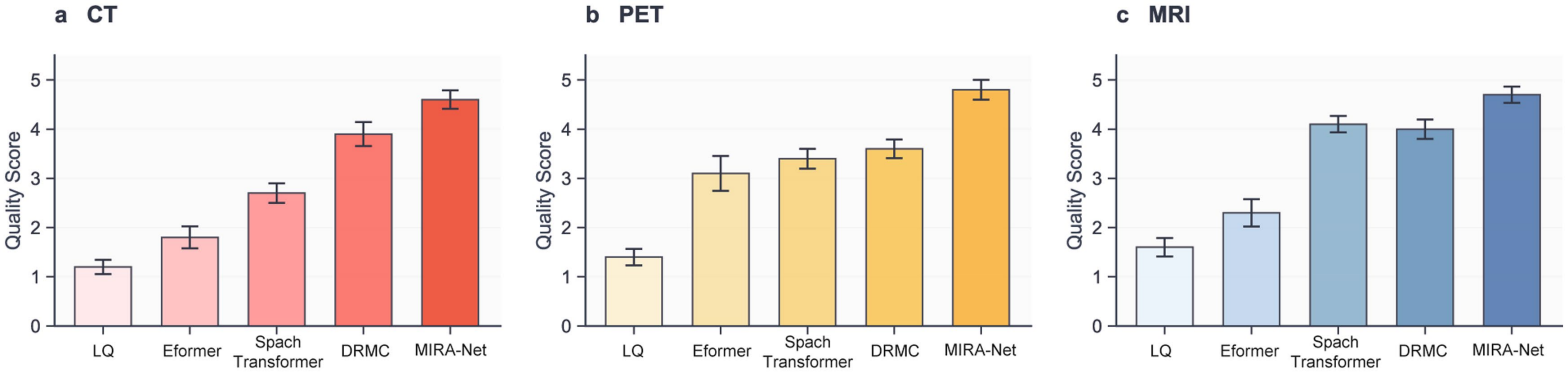
Clinical evaluation scores assigned by expert radiologists on a 5-point Likert scale. **(a)** CT modality. **(b)** PET modality. **(c)** MRI modality.

Figure 5 illustrates the results that unequivocally demonstrate the clinical superiority of the MIRA-Net model. Across all tested modalities, images restored by MIRA-Net consistently received the highest expert ratings, significantly outperforming all other methods. For CT, the composite score (mean of three readers) increased from 1.3 ± 0.4 for the low-dose input to 4.6 ± 0.3 with MIRA-Net (reader-level Wilcoxon signed-rank: *p* = 0.0007, 0.0011, 0.0008). PET showed a similar trend (1.4 ± 0.4 to 4.8 ± 0.2; *p* = 0.0006, 0.0010, 0.0007), and MRI improved from 1.6 ± 0.5 to 4.5 ± 0.3 (*p* = 0.0009, 0.0013, 0.0010; all *p* < 0.01 after Bonferroni correction). Detailed per-reader statistics are summarized in Supplementary Table S5.

## Discussion

Over the past several decades, medical imaging has become an indispensable tool in clinical practice, utilized for everything from disease diagnosis to screening (36–38). It enables clinicians to visualize organs, bones, and tissues with high precision, thereby aiding in the diagnosis and monitoring of a wide range of conditions, including cancer, cardiovascular disease, and traumatic injuries (39). However, the reliance on ionizing radiation, particularly from radioactive tracers integral to procedures like PET/CT, poses a significant health risk to patients (40). Studies have shown that the radiation dose from a single whole-body PET/CT examination can contribute to a non-trivial increase in lifetime cancer risk (41). Consequently, a critical imperative in the field is to develop methods that can restore the quality of low-dose images to a level comparable with full-dose scans, thereby drastically reducing patient exposure. To address this, deep learning models such as CycleGAN have been proposed to reconstruct standard-quality PET images from low-dose or rapidly acquired data (42). Yet, as previous research has demonstrated, artificial intelligence algorithms designed for a single, specific task often face significant performance degradation when applied to different modalities or datasets, a key limitation in versatile clinical settings (43, 44). Here, we present MIRA-Net, a unified computational framework that overcomes this limitation through an input-specific guidance mechanism. MIRA-Net not only surpasses state-of-the-art specialized models in discrete restoration tasks but, crucially, maintains this high performance when concurrently processing multiple modalities within a single unified architecture. Our validation on local clinical datasets demonstrates robust generalization across different patient populations, confirming its readiness for clinical deployment.

The cornerstone of our approach is its task-adaptive guidance mechanism, which represents a fundamental paradigm shift in unified medical image restoration. Unlike conventional single-purpose models or naive multi-task architectures, MIRA-Net leverages a dynamic instruction network to infer the modality and degradation characteristics from the input image itself, generating a unique “task instruction.” This instruction then orchestrates a fine-grained, layer-by-layer “micro-management” of feature processing via feature modulation, guiding the network to adopt the optimal strategy for the specific task at hand. This design fundamentally resolves the issue of “negative transfer” that plagues conventional unified architectures. Previous unified models, such as AirNet (45) and DRMC (35), rely on extensive parameter sharing, making them susceptible to feature-space interference between different data streams. In contrast, our instruction-guided mechanism is analogous to dynamically configuring a virtual, dedicated sub-network for each task. This preserves the model’s unified nature while enabling specialized and isolated processing strategies—a conclusion strongly supported by our all-in-one experimental results, where MIRA-Net’s performance shows negligible degradation and comprehensively surpasses all competing models. This concept aligns with the current trajectory in artificial general intelligence (46), where large models are steered by instructions or prompts to perform specific tasks (47–49), highlighting the immense potential of introducing high-level guidance mechanisms into medical image analysis. While MIRA-Net demonstrated robust performance across the board, we identified specific failure cases. As illustrated in Figure 2, in images with extremely severe noise levels or containing rare, out-of-distribution artifacts, the model’s restoration occasionally left minor residual artifacts or resulted in a subtle loss of fine-grained texture. The framework achieved its highest fidelity in CT denoising, which we attribute to the relatively uniform statistical properties of noise in CT imaging (50). In contrast, PET image restoration posed a greater challenge due to the inherent low-count statistics and Poisson noise (51), which sometimes led to a slightly lower structural similarity index compared to CT and MRI (52). In the context of MRI super-resolution, the principal challenge was the faithful reconstruction of high-frequency anatomical details without inducing spurious artifacts. Although MIRA-Net demonstrated proficient performance in this task, it addresses an inherently ill-posed problem that remains a critical area for future research.

While our study presents significant advances, we acknowledge that the experimental scope is limited to three representative modalities and restoration tasks; further validation on additional clinically relevant degradations—such as MRI motion artifacts and CT metal artifacts—remains an important direction. Conceptually, these problems can be viewed as distinct degradation operators with different frequency- and structure-dependent signatures; because ARC infers a compact instruction directly from the corrupted input and modulates feature processing throughout the network, extending MIRA-Net mainly requires task-appropriate training pair and updating the instruction dictionary to cover the new degradation regime. Second, future efforts should focus on the end-to-end integration of this high-fidelity restoration framework with downstream diagnostic AI tasks. A unified intelligent system that can simultaneously optimize image quality and improve diagnostic accuracy will pave the way for precision medicine and automated clinical decision support. Third, model interpretability and clinical trust represent critical, yet unresolved, challenges in the deployment of medical AI systems. In contrast to traditional image processing algorithms grounded in explicit mathematical formulations, deep neural networks often operate with a lack of transparency, obscuring the rationale behind their restoration decisions from radiologists. In clinical scenarios where diagnoses are directly influenced by AI-restored images, this opacity raises significant concerns about accountability, potential error propagation, and the overall confidence in diagnostic outcomes. Consequently, developing interpretable restoration frameworks is paramount for gaining clinical acceptance and securing regulatory approval.

## Conclusion

MIRA-Net provides a robust, high-fidelity solution for multi-modal medical image restoration. Its instruction-guided architecture successfully mitigates task interference, demonstrating a practical and effective pathway to reducing radiation exposure without sacrificing diagnostic quality. The framework’s superior performance across CT, PET, and MRI restoration tasks, combined with excellent clinical validation scores from expert radiologists, establishes MIRA-Net as a significant advancement in medical imaging technology. This unified approach represents a paradigm shift from single-task specialized models toward comprehensive, adaptable solutions that can address the complex demands of modern clinical workflows while maintaining the highest standards of diagnostic utility.

## Data Availability

The raw data supporting the conclusions of this article will be made available by the authors, without undue reservation.
